# Performance of an acoustic settler versus a hollow fiber–based ATF technology for influenza virus production in perfusion

**DOI:** 10.1007/s00253-020-10596-x

**Published:** 2020-04-15

**Authors:** Gwendal Gränicher, Juliana Coronel, Felix Trampler, Ingo Jordan, Yvonne Genzel, Udo Reichl

**Affiliations:** 1 Bioprocess Engineering, Max Planck Institute for Dynamics of Complex Technical Systems, Sandtorstr. 1, 39106 Magdeburg, Germany; 2 SonoSep Technologies, Waldgasse 7, 2371 Hinterbrühl, Austria; 3 ProBioGen AG, Goethestr 54, 13086 Berlin, Germany; 4 Bioprocess Engineering, Otto-von-Guericke-University Magdeburg, Universitätsplatz 2, 39106 Magdeburg, Germany

**Keywords:** Cell culture–based, Influenza, Virus, Perfusion

## Abstract

**Abstract:**

Process intensification and integration is crucial regarding an ever increasing pressure on manufacturing costs and capacities in biologics manufacturing. For virus production in perfusion mode, membrane-based alternating tangential flow filtration (ATF) and acoustic settler are the commonly described cell retention technologies. While acoustic settlers allow for continuous influenza virus harvesting, the use of commercially available membranes for ATF systems typically results in the accumulation of virus particles in the bioreactor vessel. Accordingly, with one single harvest at the end of a cultivation, this increases the risk of lowering the product quality. To assess which cell retention device would be most suitable for influenza A virus production, we compared various key performance figures using AGE1.CR.pIX cells at concentrations between 25 and 50 × 10^6^ cells/mL at similar infection conditions using either an ATF system or an acoustic settler. Production yields, process-related impurities, and aggregation of viruses and other large molecules were evaluated. Taking into account the total number of virions from both the bioreactor and the harvest vessel, a 1.5–3.0-fold higher volumetric virus yield was obtained for the acoustic settler. In addition, fewer large-sized aggregates (virus particles and other molecules) were observed in the harvest taken directly from the bioreactor. In contrast, similar levels of process-related impurities (host cell dsDNA, total protein) were obtained in the harvest for both retention systems. Overall, a clear advantage was observed for continuous virus harvesting after the acoustic settler operation mode was optimized. This development may also allow direct integration of subsequent downstream processing steps.

**Key points:**

*• High suspension cell density, immortalized avian cell line, influenza vaccine.*

## Introduction

Ever increasing demands for vaccination and gene therapy have raised the need to develop efficient large-scale manufacturing processes, including cell culture–based virus production (Kaemmerer 2018; Rappuoli and Hanon 2018). One option for intensified, rapid, and flexible biological manufacturing is high cell density perfusion cultures (Bielser et al. 2018; Chen et al. 2018). While recombinant protein production in perfusion mode has been established in the industry for many years (Konstantinov and Cooney 2015), virus production in perfusion mode is mainly pursued in academic research (Gutiérrez-Granados et al. 2018; Tapia et al. 2016). Viruses such as influenza virus (Genzel et al. 2014; Petiot et al. 2011), attenuated yellow fever virus (Nikolay et al. 2018), adenovirus (Henry et al. 2004), lentivirus (Manceur et al. 2017), and modified vaccinia Ankara virus (Vazquez-Ramirez et al. 2019) have already been investigated for intensified production in perfusion culture. Using different setups, this technology has shown clear advantages, such as a higher volumetric productivity compared with batch processes. In case of influenza pandemics, intensified cell culture–based perfusion cultures allowing a rapid and small footprint production of viruses could be of particular interest (Hegde 2015).

Membrane-based alternating tangential flow filtration (ATF) is to date the most commonly used cell retention technology for recombinant protein production such as monoclonal antibodies (Bielser et al. 2018). For virus production, however, virus particle size, half-life of infectious virions, and surface properties have to be considered, and the most suitable cell retention technology cannot be derived from the previous work with recombinant proteins. In addition, the lytic cycle of many viruses shortens the process time while increasing host cell–derived impurity levels in the supernatant.

Many cell retention devices are available for animal cell culture including ATF systems, tangential flow filtration (TFF) systems, inclined and acoustic settlers, and hydrocyclones (Patil and Walther 2018; Voisard et al. 2003). Ideally, a cell retention device should be robust, have a high cell retention efficiency while not damaging cells, allow high-yield production, be scalable to a volumetric perfusion rate of at least 1000 L/day, enable low running costs, be commercially available (eventually in single-use), and depending on process requirements, allow continuous harvesting (Voisard et al. 2003).

Due to the lytic nature of viruses and the large size of virus particles (up to 300 nm for vaccinia viruses), the use of membrane-based perfusion systems for cell retention has been shown to be challenging. Moreover, as cell culture–based production is typically divided in two phases, cell growth and virus production, optimized operation in perfusion mode aims not only for high cell concentrations but also for increasing volumetric yields. High cell concentrations further confound the mentioned factors affecting product sieving. Membrane clogging (Cortin et al. 2004) as well as unwanted virus accumulation inside the bioreactor (Genzel et al. 2014; Vazquez-Ramirez et al. 2018; Vazquez-Ramirez et al. 2019) have been reported for membrane-based perfusion processes. In contrast, continuous harvesting of recombinant proteins has been shown to improve cell-specific productivity and product quality due to a shorter residence time inside the bioreactor (Patil and Walther 2018). Additionally, total process time could be further reduced if perfusion cultures are integrated with continuous downstream processing, potentially resulting in substantial financial benefits (Bielser et al. 2018; Walther et al. 2015).

Various other cell retention systems have been developed that may also allow continuous virus harvesting. Promising results were obtained with an acoustic settler for production of influenza virus using HEK293 cells at concentrations up to 18 × 10^6^ cells/mL (Petiot et al. 2011). However, to our knowledge, no study has so far been performed to directly compare different cell retention devices regarding overall process performance, impurity levels, and product attributes for a virus production process in perfusion mode.

Here, we tested an acoustic settler and an ATF system using an AGE1.CR.pIX avian suspension cell line for influenza A virus production in perfusion mode with similar process parameters and infection conditions at concentrations up to 50 × 10^6^ cells/mL. The setup of the acoustic settler was specifically optimized for virus production and required evaluation of the cell recirculation strategy and selection of an appropriate flow rate inside the acoustic chamber. In contrast to traditional recombinant protein production (Dalm et al. 2005; Gorenflo et al. 2003), the optimization of the acoustic recirculation strategy was shown to be crucial to increase influenza virus yields.

To better assess the performance of a process, upstream and downstream aspects should be considered together (Agarabi et al. 2017; Wilson et al. 2019). Results showed similar host cell dsDNA and total protein contamination levels, fewer large-sized virus and other aggregates, and significant increase in volumetric virus productivity, when using an acoustic settler in comparison with an ATF system. Overall, results suggest that virus production in perfusion mode can lead to higher virus yields by continuous harvesting and may even allow end-to-end process integration.

## Material and methods

### Cell culture

The immortalized avian suspension cell line AGE1.CR.pIX (ProBioGen AG) was cultivated in the chemically defined medium CD-U3 (Biochrom-Merck) supplemented with 2 mM L-glutamine (Sigma), 2 mM L-alanine (Sigma), and 10 ng/mL recombinant insulin-like growth factor (LONG-R^3^ IGF, Sigma). Cells were grown in an orbital shaker (Multitron Pro, Infors HT) at 37 °C, 5% CO_2_, and 180 rpm. Baffled shake flasks with a working volume (wv) between 50 and 100 mL were used. Cells were inoculated at a viable cell concentration between 0.8 and 1.0 × 10^6^ cells/mL, and passaged every 3 to 4 days for up to 6 months.

A Biostat bioreactor (Sartorius) with a 600–700-mL wv was used to cultivate cells at larger scale in perfusion mode. The bioreactor was inoculated at a concentration between 1 and 4 × 10^6^ cells/mL. The system was agitated with a pitched-blade impeller at 150–180 rpm. Dissolved oxygen (DO) level was set to 40% and controlled through pure oxygen pulse sparging using an open tube. Carbon dioxide was used for pH control (pH 7.2). Temperature was controlled at 37 °C.

### Virus

A MDCK cell–derived virus seed (human influenza virus A/PR/8/34 H1N1, Robert Koch Institute, Amp. 3138, TCID_50_ titer: 9.9 × 10^7^ infectious virions/mL) was used for infection studies. Trypsin (Gibco, # 27250-018) was prepared in PBS with an activity of 5000 units/mL (given by the manufacturer) and added to the cell culture to facilitate virus infection. Cells were infected at a multiplicity of infection (MOI) of 10^−5^ infectious virions/cell at viable cell concentrations (VCC) of 25 × 10^6^ or 50 × 10^6^ cells/mL. The use of such a low MOI was already shown in a previous study to be optimum for high-yield influenza virus production (Lohr 2014; Jordan et al. 2016). In addition, it has to be taken into account that low MOI infections minimize the use of seed viruses required for each production run, thus facilitating large scale virus manufacturing. Trypsin was added twice to the bioreactor at 0 h post-infection (hpi) (13 units/mL) and at 12–18 hpi (15–20 units/mL) to avoid trypsin out-dilution due to perfusion as described previously (Granicher et al. 2019); the molecular weight of trypsin is low enough (23 kDa) to allow easy passage through the membrane.

### Perfusion cell culture

Based on the cellular glucose demand, a cell-specific perfusion rate of 0.06 nL/cell/day was used during the growth phase as described previously (Vazquez-Ramirez et al. 2018). Perfusion was started when the concentration exceeded 5–6 × 10^6^ cells/mL. To optimize virus production, one reactor volume was replaced with fresh medium 2–3 h before infection by increasing the perfusion rate to 8–12 reactor volumes per day (day^−1^). After infection, the perfusion was stopped for 1 h and then set to a constant perfusion rate between 1.5 and 2.0 day^−1^. The bioreactor working volume was kept constant and controlled through a scale measuring the weight of the bioreactor connected to a feed pump as previously described (Nikolay et al. 2020).

**Fig. 1:**
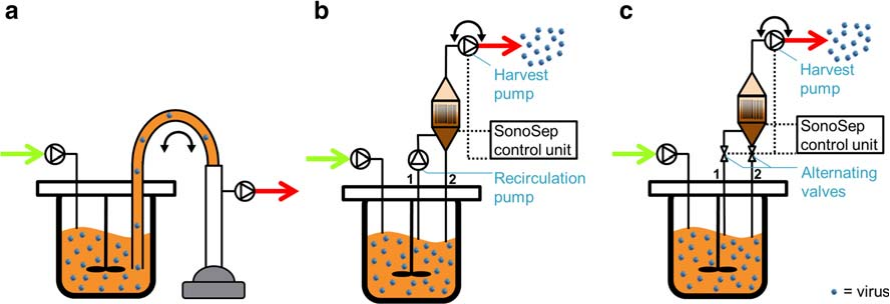
Perfusion cell culture setups with AGE1.CR.pIX cells during the influenza virus production phase. The cells were retained in the bioreactor using an alternating tangential flow filtration (A), or an acoustic settler with either a pump-based (B) or a valve-based recirculation strategy (C). The acoustic settler allowed continuous virus harvesting, which was not feasible here with the ATF system due to membrane clogging. Fresh medium was added continuously (green arrow) to feed the cells, while cell-free medium was removed (red arrow) to keep a constant bioreactor working volume

Two different cell retention devices were used for perfusion mode operation. (1) A membrane-based ATF system (ATF2, Repligen) (Fig. 1A) with a polyethersulfone (PES) hollow fiber membrane (0.2 μm pore size, 470 cm^2^, 76 fibers with 0.9 mm diameter, Spectrum). The recirculation exchange flow rate was decreased from 1.0 to 0.8 L/min compared with previous studies (Genzel et al. 2014; Vazquez-Ramirez et al. 2019) to ensure a laminar flow regime in the membranes (see “Hydrodynamic stress” section). (2) An acoustic settler (10 L acoustic chamber version, SonoSep Technologies) (Fig. 1B and C) with an acoustic power of 3 W and with a 2.1-MHz frequency was applied for all runs. The acoustic chamber was cooled through a constant air flow (at room temperature) directly on the surface of the chamber. A cell culture volume of 10 mL was exposed to the acoustic waves (total chamber volume 20 mL). The connections to the acoustic chamber had an inner diameter of 3 mm. The settler was operated in two different modes using a pump-based or a valve-based recirculation strategy.

For the pump-based recirculation strategy (Fig. 1B), cells entered the acoustic chamber through line 1 using a peristaltic pump (Watson-Marlow) using a constant recirculation rate between 3 and 8 reactor volumes day^−1^. It is important to note that in this case, two pumps are used, i.e., one for the recirculation and one for the harvesting of the cell-free medium (Fig. 1B). When exposed to the periodic acoustic waves, the cells settled and were continuously returned to the bioreactor through line 2. The cell-free supernatant was collected cycle-wise through the harvest pump. The harvest pump operated for 3 min to remove cell-free supernatant before back-flushing part of the harvested material inside the acoustic chamber at the same flow rate for 30 s. The back-flushing was done to enable high cell separation efficiencies. While back-flushing, the generation of acoustic waves was deactivated.

The valve-based recirculation strategy (Fig. 1C) was first reported by Gorenflo et al. (2003). In a first step, the cells were pumped into the chamber through line 1 and the cells were kept in the chamber due to the acoustic field, while the cell-free medium was harvested for 3 min, using the harvest pump. During this step, the valve from line 2 was kept closed. In a second step, the generation of acoustic waves was deactivated, and the harvest pump rotation was inverted to return the cells into the bioreactor through line 2 for 5–30 s at flow rates varying from 10 to 55 mL/min. The valve from line 1 was closed during the back-flushing time. The volume of harvested media per time determines the volumetric perfusion rate, and the volume of cell culture returned into the bioreactor per time determines the volumetric recirculation rate.

The cell separation efficiency (SE) was calculated as previously described (Gorenflo et al. 2005):

1 $$ \mathrm{SE}\ \left(\%\right)=\left(1-\frac{x_{\mathrm{H}}}{x_{\mathrm{Br}}}\right)\times 100 $$

where *x*_H_ is the viable or dead cell concentration in the harvest (cells/mL), and *x*_Br_ is the viable or dead cell concentration in the bioreactor (cells/mL).

In order to monitor the temperature in the acoustic settler, two sensors (80TK thermocouple module, FLUKE connected to a 73III multimeter, FLUKE) were used with the tip either on the top (outlet line) or at the bottom of the acoustic chamber (inlet/recirculation line). A culture with a viable cell concentration between 4 and 8 × 10^6^ cells/mL was used for testing this setup. In this experiment, the harvest line was redirected in the bioreactor instead of harvesting into flasks. To avoid contaminations due to the use of a sensor that cannot be autoclaved, the medium was supplemented with 0.1% (*v*/v) streptomycin (Sigma) for the testing of the setup but not for virus production (see temperature variation results, Table 1).

**Table 1:** Process conditions, cell retention efficiency, and product yields for influenza A/PR/8/34 virus using AGE1.CR.pIX cells in perfusion mode coupled with either an acoustic settler (different operation modes) or an ATF system

	ATF		Acoustic settler							
	s 1	Run 2^a^	Run 3	Run 4	Run 5	Run 6	Run 7	Run 8	Run 9	Run 10
Bioreactor working volume (mL)^b^	750^c^	800	600	670	600	600	680	600	1380	670
Recirculation strategy	-	-	Valve	Valve	Pump	Pump	Pump	Pump	Pump	Pump
Recirculation rate ([day^−1^)^b^	-	-	4.1	3.4	3.7	5.0	7.9	5.0	3.8	4.9
Max. back-flushing flow rate (mL/min)	-	-	53.4	11.2	2.8	3.2	4.8	3.0	5.5	3.6
Shear rate γ (s^−1^)	2451	3395	336	70	17	20	30	19	34	23
Net perfusion rate (day^−1^)^b^	2.1	-	1.8	2.0	2.1	1.9	1.9	1.5	1.5	2.1
*τ* in acoustic wave field (min)^b^	-	-	14	11	9	13	11	16	7	10
T range inlet line (°C)^d^	-	-	31–37	32–38	31–34	32–35	31–35	33–36	30–33	31–35 ^e^
T range outlet line (°C)^d^	-	-	38–40	37–40	39–40	40–41	39–40	42–43	37–38	39–40 ^e^
VCC at TOI (10^6^ cells/mL)	25.4	23.8	24.8	24.7	26.7	25.0	25.1	24.8	26.8	49.3
Max. VCC p.i. (10^6^ cells/mL)	37.7	23.8	35.1	30.3	36.7	34.6	32.5	27.2	32.6	69.4
Viable cell retention efficiency p.i. (%)	100.0	100.0	98.9	98.7	96.7	91.6	86.6	91.6	86.4	94.4
Dead cell retention efficiency p.i. (%)	100.0	100.0	97.7	98.6	96.1	83.2	88.8	92.5	81.0	84.3
Total number of produced virions (10^13^ virions)^f^	1.90	0.48	1.36	0.93	2.48*	2.85*	1.37	1.90	3.33*	6.93*
CSVY (virions/cell)	723	340	643	520	1124*	1371*	704	1163	1701*	1665*
*P*_v_ (10^11^ virions/L/day)^g^	5.49	1.81	5.38	2.79	7.11	9.28*	3.59	6.98^h^	16.49^h^*	13.90*

*ATF*, alternating tangential flow; *Valve*, valve-based recirculation mode; *Pump*, pump-based recirculation mode; *RV*, reactor volume; *Max.*, maximum; *τ*, mean residence time; *T*, temperature; *VCC*, viable cell concentration; *TOI*, time of infection; *p.i.*, post-infection; *CSVY*, cell-specific virus yield; *P*_*v*_, volumetric virus productivity

*Significant higher value compared to control run 1

^a^From previous study (Vazquez-Ramirez et al. 2019)

^b^Constant for the virus production phase

^c^The bioreactor was sampled 1 time with a 50-mL sampling volume p.i. The working volume was then corrected to avoid a dilution with fresh medium, and started with 800 to 750 mL working volume

^d^Determined in a separate experiment as described in the “Perfusion cell culture” section

^e^Determined from run 7 conditions (similar perfusion rate)

^f^Total number of produced virions, normalized to a bioreactor working volume of 600 mL

^g^Process time (from calculated *P*_v_) is from a starting VCC of 1.2 × 10^6^ cells/mL until time point of maximum HA titer reached

^h^Volumetric virus productivity calculated with a perfusion rate (1.5 day^−1^) lower than for the control run 1 (2.1 day^−1^) resulted in an over estimation of the *P*_v_ value

### Analytics

Viable cell concentration and percentage viability were determined using a Vi-CELL XR (Beckman-Coulter). Glucose, glutamine, lactate, and ammonium concentrations were measured using a Bioprofile 100 plus (Nova biomedical). To evaluate the metabolic state of cells, the lactate yield based on glucose consumption (*Y*_Lac/Glc_) was calculated as follows:

2 $$ {Y}_{\mathrm{Lac}/\mathrm{Glc}}=\frac{\left({C}_{\mathrm{Lac},n}-{C}_{\mathrm{Lac},n-1}\right)+{\mathrm{AC}}_{\mathrm{Lac},\mathrm{H}}\times {\mathrm{p}}_{\mathrm{H}}}{\left({C}_{\mathrm{Glc},n-1}-{C}_{\mathrm{Glc},n}\right)+\left({C}_{\mathrm{Glc},0}-{\mathrm{AC}}_{\mathrm{Glc},\mathrm{H}}\right)\times {\mathrm{p}}_{\mathrm{H}}} $$

where *C*_Lac,*n*_ is the lactate concentration at time *n* (*t*_*n*_) (mM), AC_Lac,H_ is the average lactate concentration in the harvest between *t*_*n*−1_ and *t*_*n*_ (mM), p_H_ is the perfusion ratio between *t*_*n*−1_ and *t*_*n*_ (mL perfused medium/mL working volume), *C*_Glc,n_ is the glucose concentration at time *n* (mM), *C*_Glc,0_ is the glucose concentration in the fresh medium (33 mM) (mM), and AC_Glc,H_ is the average glucose concentration in the harvest between *t*_*n*−1_ and *t*_n_ (mM).

The total number of virus particles was quantified using a hemagglutination assay, and the resulting HA titer value (in log_10_ (HA units/100 μL)) was converted into virions/mL as described previously (Granicher et al. 2019; Kalbfuss et al. 2008). The infectious virus titer was quantified with a TCID_50_ assay according to Genzel and Reichl (2007). Host cell dsDNA and total protein concentrations were measured with a Picogreen and a Bradford assay, respectively (Marichal-Gallardo et al. 2017). The distribution of large-sized virus and other aggregates was measured using a disc centrifuge (CPS DC24000 UHR disc centrifuge, CPS Instruments Inc., LA, USA) following a method described earlier (Pieler et al. 2017). Cell-specific virus yield (CSVY), volumetric virus productivity (*P*_v_), host cell dsDNA per virion, and total protein per virion were calculated using the following equations:

3 $$ {\mathrm{vir}}_{\mathrm{tot}}={\mathrm{C}}_{\mathrm{vir},\mathrm{Br}}\times \mathrm{wv}+\sum {\mathrm{AC}}_{\mathrm{vir},\mathrm{H}}\times {V}_{\mathrm{H}} $$

4 $$ \mathrm{CSVY}\kern0.5em =\frac{{\mathrm{v}\mathrm{ir}}_{\mathrm{tot},\max }}{x_{\mathrm{v},\mathrm{Br},\max}\times \mathrm{wv}} $$

5 $$ {P}_{\mathrm{v}}=\frac{{\mathrm{v}\mathrm{ir}}_{\mathrm{tot},\max }\ }{V_{\mathrm{tot}}\times {t}_{\mathrm{tot}}} $$

6 $$ \mathrm{host}\ \mathrm{cell}\ \mathrm{dsDNA}/\mathrm{virion}=\frac{C_{\mathrm{dsDNA},\mathrm{Br}}\times \mathrm{wv}+\sum {\mathrm{AC}}_{\mathrm{dsDNA},\mathrm{H}}\times {V}_{\mathrm{H}}}{{\mathrm{vir}}_{\mathrm{tot}}} $$

7 $$ \mathrm{total}\ \mathrm{protein}/\mathrm{virion}=\frac{C_{\mathrm{tProt},\mathrm{Br}}\times \mathrm{wv}+\sum {\mathrm{AC}}_{\mathrm{tProt},\mathrm{H}}\times {V}_{\mathrm{H}}\ }{{\mathrm{vir}}_{\mathrm{tot}}} $$

where vir_tot_ is the total number of virions produced (−), C_vir,Br_ is the virus particle concentration in the bioreactor (virions/mL), wv is the bioreactor working volume (mL), AC_vir,H_ is the average virus particle concentration in the harvest between *t*_*n*−1_ and *t*_*n*_ (virions/mL), *V*_H_ is the harvested volume between *t*_*n*−1_ and *t*_*n*_ (mL), *x*_v,Br,max_ is the maximum concentration of viable cells in the bioreactor obtained until time point of maximum vir_tot_ (cells/mL), *V*_tot_ is the total volume of medium spent including cell growth phase until time point of maximum vir_tot_ (mL), *t*_tot_ is the time from bioreactor inoculation until maximum vir_tot_ (h), *C*_dsDNA,Br_ is the dsDNA concentration in the bioreactor (μg/mL), AC_dsDNA,H_ is the average dsDNA concentration in the harvest between *t*_*n*−1_ and *t*_*n*_ (μg/mL), *C*_tProt,Br_ is the total protein concentration in the bioreactor (μg/mL), and AC_tProt,H_ is the average total protein concentration in the harvest between *t*_*n*−1_ and *t*_*n*_ (μg/mL).

Contamination levels for host cell dsDNA/virion and total protein/virion were calculated to assess whether continuous virus harvesting has an advantage compared to ATF mode for subsequent downstream processing. To allow a comparison of vir_tot_ calculated for cultivations with different working volumes, runs were normalized to 600 mL wv.

### Hydrodynamic stress

To describe the different hydrodynamic stress conditions for the ATF system and the acoustic settler, the shear rate (γ) was estimated assuming laminar flow conditions in a cylinder based on the Reynolds number.

8 $$ \mathit{\operatorname{Re}}=\frac{\uprho \times v\times L}{\mu } $$

9 $$ \gamma =\frac{4\times Q}{n\times \pi \times {R}^3} $$

where ρ is the fluid density (g/L), *v* is the velocity of the fluid (m/s), *L* is the characteristic length (m), μ is the dynamic viscosity of the fluid (Pa × s), *Q* is the volumetric recirculation rate (m^3^/s), *n* is the number of fibers (for an ATF membrane) (−), and *R* is the internal radius of the recirculation tube (m).

The volumetric recirculation rate was determined following the exchange flow rate for the ATF system (between 0.8 and 1.0 L/min). For the acoustic settler, the maximum back-flushing flow rate was taken to calculate γ.

### Statistical analysis

A Student’s *t* test was applied for statistical analysis using the Origin software with *p* values lower than 0.05 considered as significant.

## Results

In order to assess the impact of the cell retention device and the recirculation strategy (described in the “Perfusion cell culture” section) on the influenza virus production, perfusion cell cultures using similar infection conditions but with different recirculation strategies and recirculation flow rates were carried out. The corresponding process conditions are summarized in Table 1. In addition, cell growth before the infection phase was evaluated. Analytics comprised on cell concentrations, virus titers, retention efficiencies, harvest volumes, impurity levels, and the distribution of large-sized virus and other aggregates.

### Cell growth behavior

Efficient perfusion cultures using AGE1.CR.pIX cells, which are characterized by short doubling times (*t*_d_), high viabilities, and high cell concentrations (up to 50 × 10^6^ cells/mL), were described previously for cultivations with bioreactors coupled to an ATF device (Coronel et al. 2019; Genzel et al. 2014; Vazquez-Ramirez et al. 2018; Vazquez-Ramirez et al. 2019). Before evaluating virus yields and productivity, the growth performance of AGE1.CR.pIX cells was assessed for VCCs between 25 and 55 × 10^6^ cells/mL using an acoustic settler.

**Fig. 2:**
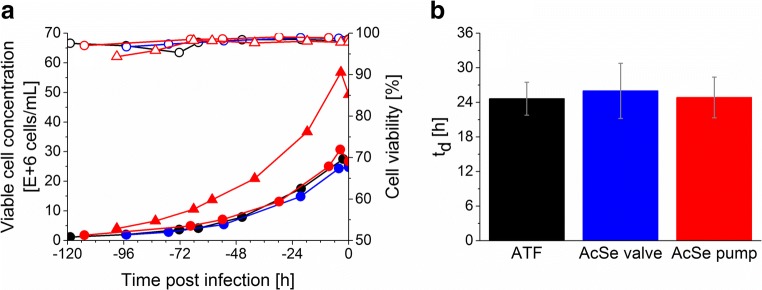
Growth of AGE1.CR.pIX cells cultivated in perfusion mode using different cell retention technologies and recirculation strategies. **a** Viable cell concentration (filled symbols) and cell viability (empty symbols) of one representative ATF run (run 1) (black circle), one representative run for the acoustic settler with valve-based recirculation (AcSE valve, run 4) (blue circle), and two representative runs for the acoustic settler with pump-based recirculation (AcSE pump, run 5 (red circle), and run 10 (red triangle)). **b** Cell population doubling time (*t*_d_) calculated during the cell growth phase in perfusion mode (average between each sampling time point for each run ± standard deviation). The values correspond to run 1 for ATF (black), runs 3 and 4 for the acoustic settler with valve-based recirculation (blue), and runs 5 and 10 for the acoustic settler with pump-based recirculation (red). A CSPR of 0.06 nL/cell/day was applied for every perfusion run. Detailed operation conditions in Table 1

A cell viability above 98% for VCCs between 10 and 55 × 10^6^ cells/mL was obtained using the acoustic settler in either pump- or valve-based recirculation mode (Fig. 2a). Small variations of *t*_d_ were observed for the different perfusion setups (Fig. 2b), but differences were statistically not significant (*t* test). All acoustic settler runs showed cell retention efficiencies before infection above 98% (data not shown).

### Influenza virus production

#### Process performance

Two recirculation strategies and various recirculation rates were tested for acoustic settler operation. Following the cell growth phase, the cells were infected with influenza virus at a VCC of at least 25 × 10^6^ cells/mL. Different product yields were obtained for the cultivations, namely CSVY and *P*_v_ (Table 1). For all runs (except ATF run 2 from a previous study), the same MOI, trypsin activity, perfusion rate, and VCC at TOI were used. Run 10 differed as a higher VCC at TOI was tested. The main differences were therefore related to the recirculation strategy and recirculation rate of the acoustic settler.

CSVYs and *P*_v_s over 1124 virions/cell and 7.11 × 10^11^ virions/L/day were obtained, respectively, for cultivations using an acoustic settler with a pump-based recirculation rate between 3.7 and 5.0 day^−1^ (runs 5, 6, and 9). Under these conditions, higher yields (about 1.5-fold higher) were obtained with the acoustic settler compared with the ATF system (run 1; CSVY = 723 virions/cell, *P*_v_ = 5.49 × 10^11^ virions/L/day). For higher pump-based recirculation rates (7.9 day^−1^, run 7), a lower CSVY (704 virions/cell) and a lower *P*_v_ (3.59 × 10^11^ virions/L/day) were observed, compared with the aforementioned cultivations with lower recirculation rates. In addition, acoustic settler perfusion runs using the valve-based recirculation mode (runs 3 and 4) resulted in lower yields compared with run 5 using a similar recirculation rate (but in pump-based recirculation mode). The valve-based recirculation strategy achieved the highest cell retention efficiency (over 98%, runs 3 and 4), whereas a slightly reduced cell retention efficiency after infection was observed with increased pump-based recirculation rate (from 3.7 to 7.9 day^−1^). Virus accumulation inside of the bioreactor was observed for the ATF run as only 7.5% of vir_tot_ was harvested through the membrane. For all AS runs, no virus accumulation was observed.

Based on the perfusion parameters of runs 5 and 6, a VCC of 50 × 10^6^ cells/mL (at TOI) was achieved in virus production (run 10, Table 1). The CSVY of run 10 was not decreased compared with other runs using the same recirculation mode and same perfusion rate but infected at lower cell concentrations (runs 5 and 6). A *P*_v_ increase of 2.5 was obtained when comparing run 10 with the ATF run 1.

The virus production phase was evaluated as before by VCC and cell viability. In addition, as an indicator for cell metabolism and cell stress, lactate release and *Y*_Lac/Glc_ yield of perfusion runs 1 and 3–7 (Table 1) was monitored.

**Fig. 3:**
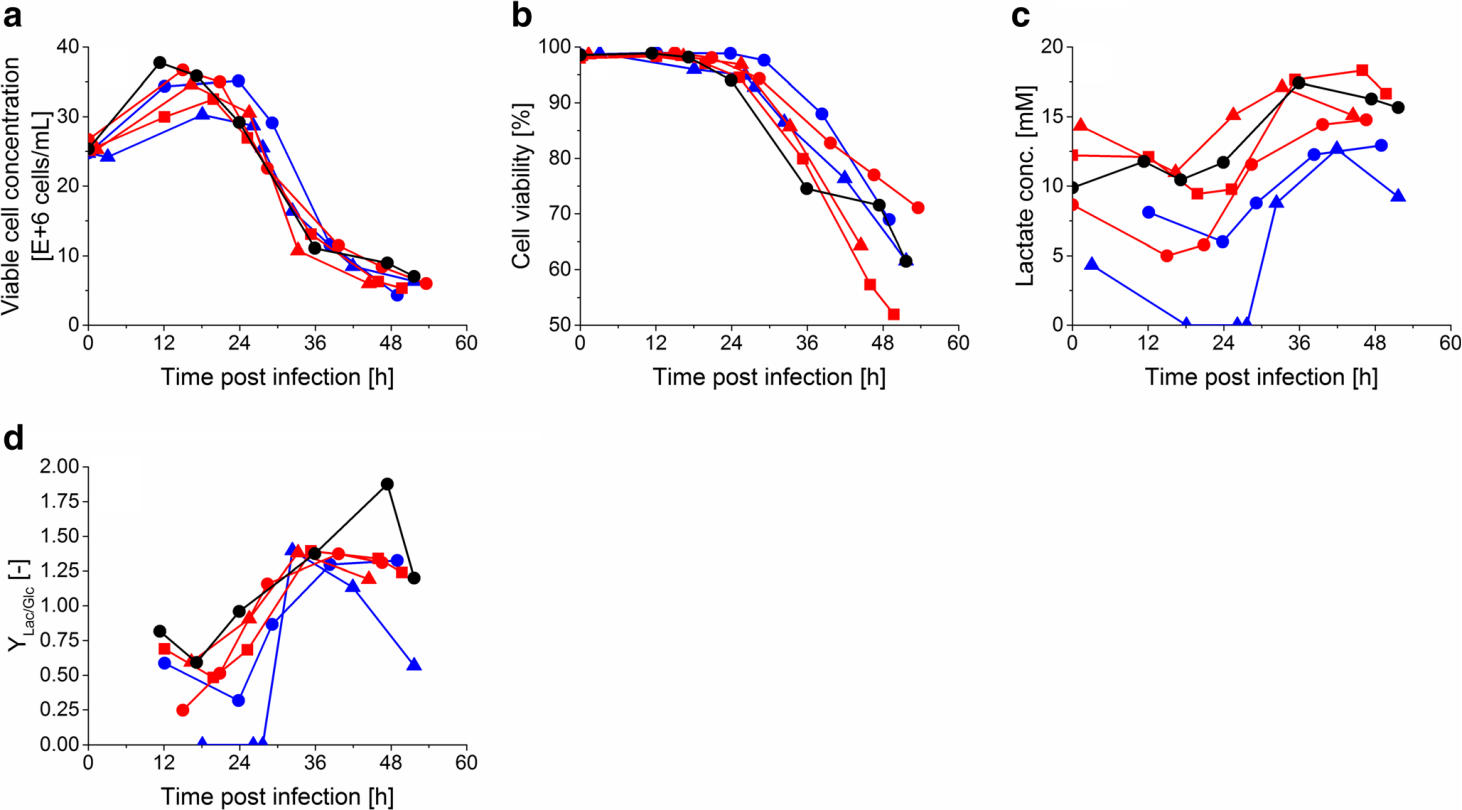
Influence of acoustic settler operation on viable cell concentration, viability, and lactate metabolism during the influenza A/PR/8/34 virus production phase with AGE1.CR.pIX cells. (**a**) Viable cell concentration, (**b**) cell viability, (**c**) lactate concentration in the bioreactor supernatant. (**d**) *Y*_Lac/Glc_ yield. Run 1 (black circle): performed with the ATF system. Run 3 (blue circle) and run 4 (blue triangle): performed with the valve-based recirculation mode of the acoustic settler. Run 5 (red circle), run 6 (red triangle), and run 7 (red square): performed with the pump-based recirculation mode of the acoustic settler. Detailed operation conditions in Table 1

Similar viable cell growth and cell viability profiles were observed for run 1 and runs 3–7 (Fig. 3a, b). In all cultivations, a VCC of at least 30 × 10^6^ cells/mL was reached and cell viability was maintained above 95% during the first 24 hpi. A higher *Y*_Lac/Glc_ and lactate concentration was observed with perfusion in a pump-based recirculation mode (runs 5–7) compared with the runs 3 and 4 using a valve-based recirculation mode (Fig. 3c, d) during at least the first 24 hpi. During the first 36 hpi, the ATF run showed similar *Y*_Lac/Glc_ yields and lactate concentration compared with the runs with pump-based recirculation (using the acoustic settler).

#### Process-related impurities

As previously mentioned in the “Perfusion cell culture” and “Process performance” sections, the choice of the cell retention device is expected to have an impact on influenza virus production in perfusion mode. While the acoustic settler allows continuous harvesting, the membrane-based cell retention technology tends to lead to accumulation of the virus inside the bioreactor. To assess which cell retention device would facilitate downstream processing, the two main process-related impurities, namely, host cell dsDNA and total protein concentrations were measured during the virus production phase.

**Fig. 4:**
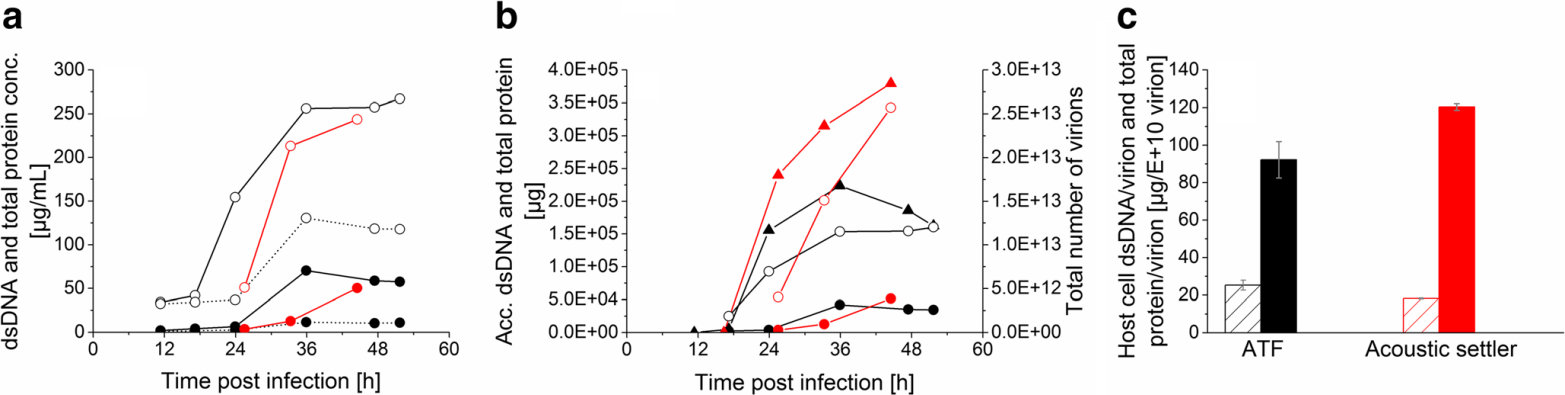
Host cell dsDNA and total protein impurity levels during influenza A/PR/8/34 virus production phase in AGE1.CR.pIX cells in perfusion mode using an ATF system (run 1, black) or an acoustic settler with pump-based recirculation (one representative optimized run, run 6, red). **a** Host cell dsDNA concentration (black circle) and total protein concentration (white circle). Dashed lines represent additional data from the ATF permeate line. **b** Accumulated dsDNA (black circle), accumulated total protein (white circle), and total number of produced virions over time (black triangle). **c** Host cell dsDNA per virion (striped columns) and total protein per virion (filled columns) at optimum harvest time point (average ± standard deviation of technical duplicates). For the ATF cultivation, the bioreactor content was harvested at 36 hpi. When using the acoustic settler, virions from the bioreactor were also collected at the optimum harvest time point which corresponded to 45 hpi. Detailed operation conditions in Table 1

In the bioreactor, higher host cell dsDNA and total protein concentrations were measured in comparison with the permeate for the ATF system (Fig. 4a). In addition, a decline in the total number of accumulated virions was observed after 36 hpi (Fig. 4b). In contrast, a higher accumulation of total protein and a higher total number of produced virions were observed for the cultivation using an acoustic settler (Fig. 4b). Finally, host cell dsDNA as well as the total protein impurity levels per virion were similar at the respective optimum time of harvest (Fig. 4c).

#### Impact of the cell retention device on infectious virus titers and virus aggregation

Infectious titers and extend of aggregation of virus particles can be important product quality attributes in the design of a vaccine production process. Infectivity is of particular interest in case of live-attenuated influenza vaccine manufacturing. Virus aggregation also influences infectivity and, even more important, may negatively affect virus recovery in subsequent downstream processing.

**Fig. 5:**
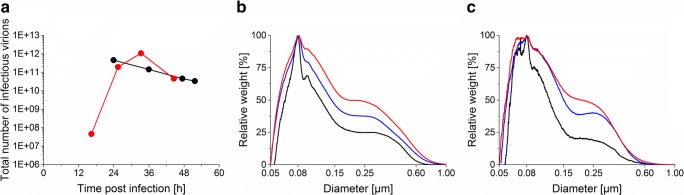
Infectious titer of influenza A/PR/8/34 virus and size distributions during influenza A/PR/8/34 virus production phase in AGE1.CR.pIX cells in perfusion mode using either an ATF system or an acoustic settler with pump-based recirculation. (**a**) Total number of infectious influenza virus particles produced using an ATF system (run 1, black circle) or an acoustic settler (one representative optimized run, run 6, red circle). Size distributions of run 1 (ATF, **b**) and run 6 (acoustic settler, **c**). All samples were measured from the crude bioreactor supernatant. For graph **b**, black, blue, and red lines correspond to 24, 36, and 47 hpi, respectively. For graph **c**, black, blue, and red lines correspond to 25, 33, and 45 hpi, respectively. Detailed operation conditions in Table 1

A similar total number of infectious virions (sum of virions in the vessel and in the harvest) were obtained for both the ATF system and the acoustic settler (Fig. 5a, difference within the error range of the TCID_50_ assay). For both cell retention devices, the number of infectious virions decreased after maximum values were reached. An aggregation increase over time of viruses and other large molecules was observed between 24 and 48 hpi for both systems in either the bioreactor (ATF, Fig. 5b) or the harvest collected via continuous harvesting (acoustic settler, Fig. 5c). When using the acoustic settler, a higher amount of small debris (under 0.08 μm) and a lower amount of large-sized debris (above 0.60 μm) were observed between 24 and 48 hpi compared with operation with the ATF system (Fig. 5b, c).

## Discussion

High cell density cultivation in perfusion mode has advantages for efficient production of viruses, viral vectors, or recombinant proteins. While various options exist, the use of acoustic settlers has already been reported to be efficient for many virus production processes (Gutiérrez-Granados et al. 2018). However, they can be set up in different modes of operation, and no results was available regarding the design and optimization of virus production processes performed at concentrations as high as 70 × 10^6^ viable cells/mL.

Compared with recombinant protein production, cell metabolism, cell viability, level of impurities, and overall culture viscosity (due to cell lysis and death) change drastically after infection, which can have a significant impact on cell retention and virus harvesting. Previous studies using mammalian cell cultures for recombinant protein production have shown that the recirculation mode (Gorenflo et al. 2003) and the recirculation rate (between 2 and 15 day^−1^) (Dalm et al. 2005; Gorenflo et al. 2005; Gorenflo et al. 2002; Shirgaonkar et al. 2004) influence the cell retention efficiency, but do not impact cell viability or productivity. Similar doubling times and high viabilities before infection were also observed for the cell growth phase in the present study comparing an acoustic settler with different recirculation strategies and an ATF system. Furthermore, the *t*_d_ values obtained with the acoustic settler in this study were in agreement with values reported previously for AGE1.CR.pIX cells cultivated in perfusion mode with an ATF system operated at higher exchange rates (between 28 and 41 h) (Coronel et al. 2019; Vazquez-Ramirez et al. 2019).

Regarding the virus infection phase, however, the recirculation strategy of the acoustic settler turned out to have a major impact on productivity, even for a process time as short as 48 h. Indeed, with recirculation rates above 7 day^−1^, the CSVY decreased by a factor of 1.7 compared with recirculation rates between 3.7 and 5.0 day^−1^. One reason could be an increase in mechanical stress on cells from the peristaltic pump. As reported for other cell lines, peristaltic pumps can impact cell viability (Blaschczok et al. 2013) or cell-specific productivity even at recirculation rates as low as 1.4 day^−1^ (Merten 2000). Furthermore, higher influenza virus yields have already been shown for low hydrodynamic stress conditions in an orbitally shaken bioreactor without gas sparging and impeller agitation for AGE1.CR.pIX cells (Coronel et al. 2019). Furthermore, a cell line may be more sensitive to mechanical stress after infection due to metabolic changes and the addition of the protease trypsin that activates influenza virus infectivity but can also interfere with plasma membrane integrity. For example, Cortin et al. (2004) reported a higher sensitivity to hydrodynamic stress for human 293S cells infected with adenovirus that were cultivated at concentrations of only 8 × 10^6^ cells/mL using a membrane-based perfusion system. The use of recirculation rates between 3.7 and 5.0 day^−1^ with perfusion rates of 1.5–2.0 day^−1^ should also be feasible in large scale production. For example, Gorenflo et al. (2002) reported a 96% cell retention efficiency for CHO cells and production of recombinant proteins at a volumetric perfusion rate of 200 L/day and a volumetric recirculation rate of 600 L/day. In this case, a ratio of 3 (600/200) was used as in our study.

Regarding the acoustic settler, lower CSVY and *P*_v_ were observed for the valve-based recirculation strategy (runs 3 and 4) compared with the pump-based recirculation strategy (run 5), although a similar recirculation rate was used (Table 1). This was an unexpected result as the valve-based recirculation strategy avoids the use of a recirculation pump, which should reduce the mechanical stress on cells. However, for perfusion operation using the valve-based recirculation, lower *Y*_Lac/Glc_ and lactate levels consistent with lower stress levels in the culture were also observed (Fig. 3). Furthermore, oscillations in process conditions can influence (positively or negatively) the productivity of mammalian cell cultures (Lara et al. 2006; Spadiut et al. 2013). Cells in the acoustic settler are exposed to fluctuations of the DO level and temperature (Dalm et al. 2005; Drouin et al. 2007). In our case, a higher maximum temperature was observed for the operation in valve mode with a similar recirculation rate and perfusion rate compared with pump-based operation (runs 3 and 4 vs runs 5 and 6, Table 1). These physical observations may explain the negative impact on productivity of the recirculation strategy. Drouin et al. (2007) reported a decrease in the productivity for the investigated recombinant protein when the temperature in the upper part of the acoustic settler was oscillating up to 38.5 °C. In our case, temperatures as high as 40 °C were reached in the upper part of the acoustic chamber during the virus production phase independent of the recirculation strategy. However, temperatures of 37 °C and 35 °C were measured in the lower part of the acoustic chamber for the valve-based and pump-based recirculation strategy, respectively (Table 1). A higher temperature on the lower part of the acoustic settler (containing the aggregated cells) could have negatively influenced the virus production when recirculating the cells in the acoustic settler with the valve-based mode. Temperatures above 37 °C might be detrimental for cell metabolism and virus stability. In addition to the impact of temperature, Dalm et al. (2005) reported DO levels dropping to zero in the acoustic settler for recirculation rates of up to 6 day^−1^. This might be detrimental for the cell-based virus production as well. Higher *P*_v_ and CSVY observed in run 9 (Table 1) with temperatures limited to 38 °C on the upper part of the acoustic chamber suggest that virus production using acoustic settlers could be further improved (by more than a factor 1.5) compared with the ATF system. Possible options would be either the use of a smaller acoustic chamber and a more efficient cooling system or operation at a higher volumetric perfusion rate (> 1.2 L/day).

An increase by a factor of at least 1.5 for *P*_v_ and CSVY was observed in the most successful perfusion runs with the acoustic settler compared with cultivations performed with the ATF system (Table 1). This increase could be explained by several factors. Most likely, the continuous removal of virions resulted in lower infectivity losses due to a shorter exposition to proteases released from lysed cells. In addition, reduced levels of accumulated host cell proteins that can potentially inhibit virus production may also play a role. Moreover, acoustic settlers also allow for the selective removal of dead cells over viable cells, which might be beneficial for virus production as it reduces the risk of virus degradation by protease activity and the accumulation of signaling molecules and unspecific inhibitors of virus replication. Lower shear rates during infection may also contribute to better yields. With a calculated γ < 340 s^−1^, the conditions in the acoustic settler seem to be superior to the ATF system (γ = 2451 s^−1^, Table 1). Although various mammalian and insect cell lines were shown to survive hydrodynamic stress up to γ = 3000 s^−1^ (Maiorella et al. 1991) or γ = 4000 s^−1^ (van Reis et al. 1991), values as low as γ = 620 s^−1^ were shown to be harmful for 293S cells after adenovirus infection (Cortin et al. 2004).

The use of acoustic settler for influenza virus production when cells were infected at concentrations up to 50 × 10^6^ cells/mL (run 10) was also possible. A maximum VCC of approximately 70 × 10^6^ cells/mL was reached post-infection. In this run, a high CSVY of 1665 virions/cell was obtained. For infections at 25 × 10^6^ cells/mL (for example runs 5 and 6) CSVYs of 1124 to 1371 virions/cell were reached. The total amount of produced virions and *P*_v_ were, by consequence, also increased in run 10. This suggests that the *P*_v_ and the total amount of produced virions per bioreactor run can be even further increased if higher cell concentrations can be achieved after the growth phase.

Interestingly, for all the runs with the highest CSVYs (runs 6, 9, and 10), a difference of at least 5% was observed between the viable and the dead cell retention efficiencies after infection (Table 1). This suggests that a selective removal of dead cells over viable cells through the acoustic settler that increases the percentage of viable cells in the cultivation vessel might be an additional option for improving virus titers. For example, various studies have shown an increased product yield when increasing the cell viability (Antoni et al. 1995; Palomares et al. 2000).

Similar process-related impurity levels (host cell dsDNA and total protein per virion) were observed in the harvest of the ATF and the acoustic settler perfusion cultures (Fig. 4c). For the ATF run, higher impurity concentrations were detected inside the bioreactor compared with the permeate line (Fig. 4a). As expected from previous work (Granicher et al. 2019), in addition to enrichment of viruses, dsDNA and proteins also accumulate inside the bioreactor in membrane-based perfusion cultures. In contrast to the acoustic settler, the harvest from ATF cultures is taken directly from the bioreactor, which requires an additional clarification step for subsequent virus purification. In contrast, an acoustic settler has a higher flexibility in terms of the optimum harvest time point. In addition, as the virus is harvested continuously through the acoustic chamber, there is a lower risk of virus degradation (as seen in Fig. 4b for the ATF).

Continuous harvesting is, in particular, attractive due to the inherent instability of some viruses. For example, a loss of influenza A virus infectivity by four orders of magnitude was observed after an incubation time of 48 h at 37 °C (Petiot et al. 2011). In our study, perfusion rates of 1.5 to 2.0 day^−1^ were used with the acoustic settler that corresponds to relatively short residence times of virions in the bioreactor. A higher total amount of infectious virions was obtained (TCID_50_ assay) for the cultivation with the acoustic settler (4.6 × 10^11^ for the ATF vs 10.8 × 10^11^ for the acoustic settler, Fig. 5a). However, this difference might be negligible as it is close to the assay error (about ± 0.3 log, Genzel and Reichl 2007). Therefore, operation at a perfusion rate of 2 day^−1^ seems not sufficient to avoid infectious virion losses. To a certain extent, a partial reduction of virus infectivity for cultivations using an acoustic settler might also be expected due to the temperature increase in the acoustic chamber. Finally, in the case of influenza virus production with the vast majority of licensed vaccines using killed virus, the titer calculated from the HA assay (the total number of all virus particles) is of higher importance. For live vaccines using attenuated polio, yellow fever, or measles virus, losses in the infectious titer would be more critical.

Virus aggregation and formation of other large-sized aggregates was observed for later time points of infection for cultivations with the ATF system and the acoustic settler. While profiles look similar, a lower amount of aggregates larger than 0.6 μm was observed for cultivations performed with the acoustic settler (Fig. 5b, c). Perfusion rates higher than 2 day^−1^ might be considered to limit the formation of aggregates in the bioreactor and to facilitate subsequent purification steps. A further reduction of aggregates is desirable because virus aggregation typically results in lower process yields, and high levels of protein aggregates can result in a strong but unwanted induction of immune responses (Ratanji et al. 2014; Rosenberg 2006; Wang et al. 2012).

Disadvantages regarding the use of ATF systems in viral vaccine production due to an accumulation and eventual degradation of virions inside of the bioreactor may be alleviated by selection of membranes that are better suited for this type of application or specifically designed for virus production processes. Similarly to what was tested for membrane-based perfusion culture in recombinant protein production (Esclade et al. 1991; Mercille et al. 1994; Pinto et al. 2019), different membrane chemistries, pore sizes, and properties such as hydrophobicity and surface charge should be characterized regarding product sieving and membrane fouling. For example, Genzel et al. (2014) tested various polysulfone and polyether sulfone membranes for influenza virus production in perfusion mode. However, even with pore sizes up to 0.5 μm, virus retention inside of the bioreactor could not be prevented. Clearly, virus harvesting through a membrane is more challenging than establishment of membrane-based perfusion processes for recombinant proteins, taking into account in particular the relatively large size of virions and their lytic replication cycle resulting in significant changes in composition and viscosity of supernatants. However, one obvious advantage of ATF systems is their availability for single use manufacturing, which can facilitate scale-up and process validation.

In conclusion, a scalable perfusion process based on an acoustic settler with concentrations above 50 × 10^6^ cells/mL was developed for continuous harvesting of influenza viruses. For acoustic settlers, the recirculation strategy, recirculation rates, and temperature elevation in the inner acoustic chamber were found to have a large influence on the CSVY and the *P*_v_. Virus yields were 1.5- to 3.0-folds higher for the acoustic settler compared with ATF. Acoustic settlers have been shown to be scalable to volumetric perfusion rates of at least 200 L/day (Gorenflo et al. 2002), and one manufacturer (Applikon Biotechnology) commercializes acoustic settlers for volumetric perfusion rates of up to 1000 L/day. Our results suggest that the acoustic cell retention technology could be applicable for the production of viral vaccines even at large scale. Continuous harvesting may be especially beneficial for the production of live vaccines or viral vectors for gene therapy where prevention of infectivity losses due to degradation of particles is crucial for product quality. Furthermore, the establishment of continuous harvesting schemes using acoustic settlers might help to establish fully integrated vaccine production processes.
